# De novo mutation of *CYBB* gene in a boy presenting as intra-abdominal infection of Burkholderia contaminans: a case report

**DOI:** 10.1186/s13052-022-01246-1

**Published:** 2022-04-01

**Authors:** Qianqian Zhao, Jing Yin, Jijun Ma, Xiaoxue Liu, Jiawen Wu, Chongwei Li

**Affiliations:** Department of Pediatric Immunology, Tianjin Children’s Hospital (Tianjin University Children’s Hospital), Tianjin, China

**Keywords:** Chronic granulomatous disease (CGD), *CYBB*, De novo, Mutation, Abdominal infection, *Burkholderia contaminans*

## Abstract

**Background:**

Chronic granulomatous disease (CGD) is an inborn error of immunity. It is characterized by recurrent bacterial or fungal infections, including infections by *Burkholderia species*. This is due to respiratory burst dysfunction of phagocytes. Currently, there is no report on *Burkholderia contaminans (B. Contaminans)* infection in children with CGD.

**Case presentation:**

We present a previously healthy, 17-month-old Chinese boy infected with *B. Contaminans* in the intra-abdominal regions. Immunological screening, including assessment of cellular immunity and humoral immunity did not yield conclusive results. The level of nicotinamide adenine dinucleotide phosphatase (NADPH) activity was decreased and whole-exome sequencing identified a de novo mutation in the *CYBB* gene.

**Conclusions:**

For specific pathogens such as *B. Contaminans*, immune assessment should be carried out even if there is no positive medical history or specificity in basic immunity screening.

## Background

Chronic granulomatous disease (CGD) is a rare inborn error of immunity with an incidence of 1/250000 to 1/200000 and high mortality rate [1]. Defects in genes encoding various components of the nicotinamide adenine dinucleotide phosphate (NADPH) oxidase complex are associated with a dysfunctional respiratory burst, which decreases the ability of phagocytes to kill catalase-positive bacteria and fungi [2]. Mutations in *CYBB*, *CYBA*, *NCF1*, *NCF2*, *NCF4*, and *CYBC1* genes have been associated with CGD [3]. About 70% of CGD cases are caused by defects in the *CYBB* gene, which is located on the short arm of the X chromosome (Xp21.1-p11.4). The gene encodes the *gp91phox* subunit that causes X-linked CGD (XLR-CGD). Defects in genes encoding the other NADPH oxidase subunits, including *p22phox, p47phox, p67phox,* and *p40phox,* lead to autosomal recessive CGD (AR-CGD). *CYBB* gene mutations include deletions, insertions, nonsense mutations, missense mutations, or splicing errors. Most CGD cases associated with *CYBB* gene mutations are hereditary and about 10 to 15% of cases are due to de novo mutations [4].

Patients with CGD mainly present with recurrent bacterial or fungal infections, granuloma formation, *BCG-osis*, inflammatory manifestations, and autoimmune phenomena (cutaneous lupus erythematosus, idiopathic thrombocytopenia or juvenile idiopathic arthritis). *Burkholderia cepacia* (*B.cepacia*) is the most widely known as a susceptible pathogen of CGD. However, *B. Contaminans* infection in children with CGD has not been reported.

Here, we describe a case of a 17-month-old boy infected with *B. Contaminans* in the intra-abdominal region, who was later diagnosed with XLR-CGD, characterized by de novo nonsense mutation in exon 6 of the *CYBB* gene (c. 603C > A).

## Case presentation

**Fig. 1:**
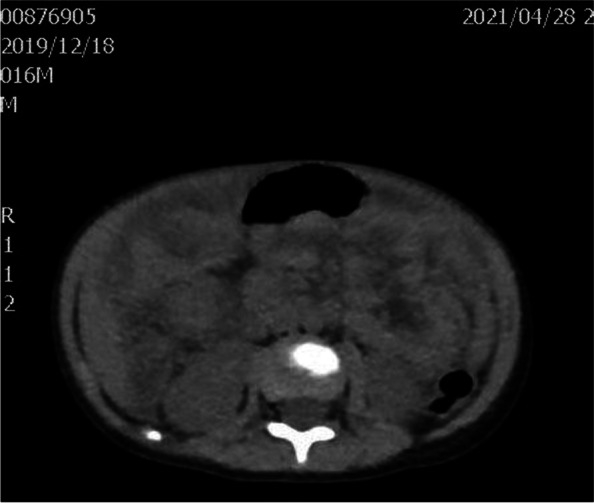
Abdominal CT scan showed localized intestinal wall thickening in the right upper abdominal cavity, thickening of the right upper abdominal omentum, extensive lymphadenopathy and ascites

**Fig. 2:**
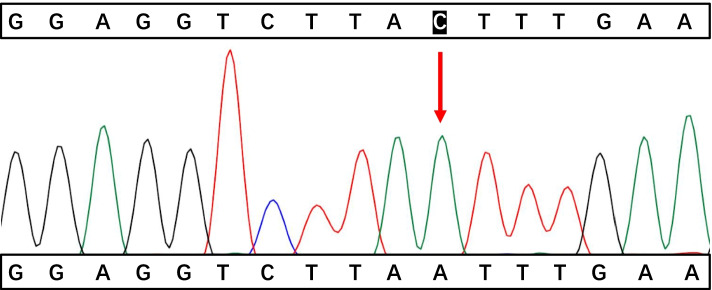
Electropherogram showing the position of the variant (NM_000397.3; c.603C > A position p. Tyr201*) in CYBB gene in the patient in a healthy control and the family members

**Fig. 3:**
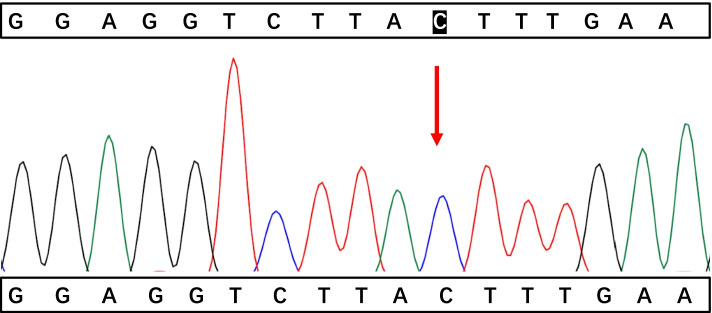
Electropherogram of the patient’s mother showing normal

**Fig. 4:**
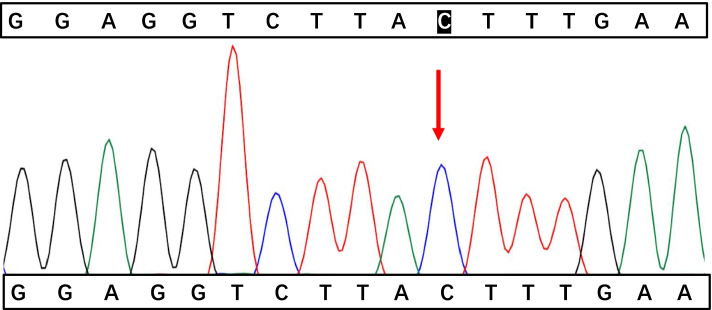
Electropherogram of the patient’s father showing normal

A 17-month-old boy was hospitalized on April 27, 2021, due to swelling in the right scrotum for seven days. He was previously healthy and has a healthy, 11-year-old brother. There was no family history of immunodeficiency or recurrent infections. Routine immunization with Bacillus Calmette- Guerin(BCG) was administered without any untoward consequences. Physical examination showed painless bilateral cervical lymphadenopathy (about 1.0cm in diameter), a normal BCG scar, no cardiopulmonary abnormalities, a soft palpable abdomen without tenderness with a circumference of 54cm, no skin rash, and an enlarged right scrotum. Routine blood tests showed moderate anemia(hemoglobin 82g/L, MCV63.2fL, MCH 19.0pg, MCHC 301g/L), leukocytosis (23.56×10^9^/L), neutrophils 57%, lymphocytes 33%, monocytes 10%, and thrombocytosis (523×10^9^/L). Other tests showed increased erythrocyte sedimentation rate (ESR 53 mm/h, normal value<20 mm/h), C-reactive protein (CRP 16.36 mg/dL,normal value<0.8mg/dL), procalcitonin (PCT1.72ng/mL, normal value<0.05ng/mL), and D-Dimer (D-Dimer 13460ng/mL, normal value<550 ng/mL). Albumin, ferritin, lactate dehydrogenase (LDH), alanine aminotransferase (ALT), aspartate aminotransferase (AST), triglycerides and fibrinogen were within the normal range, which did not meet HLH diagnostic criteria. Serum IgG and IgA levels were slightly increased to 1082 mg/dL (reference 453-916mg/dL) and 115 mg/dL (reference 21-100mg/dL) respectively,while IgM and IgE levels were normal. A flow cytometric analysis of lymphocyte subsets showed CD3 ^+^cells of 45.55% (reference value 53.88-72.87%), CD3^+^CD8^+^cells of 14.91% (reference value 19-32.51%), CD3^+^CD4^+^cells of 26.01% (reference value 24.08-42.52%), CDl9^+^cells of 44.77% (reference value 13.23-26.39%), CDl6^+^CD56^+^cells of 8.95% (reference value7.21-20.9%), and CD4/CD8 1.74 (reference value 0.90-2.13). Epstein-Barr virus and cytomegalovirus DNA detection and antibodies (IgG and IgM) in serum were negative. Further, tuberculin skin test and interferon-gamma release assay (IGRA) were negative. In addition, blood, urine, and stool culture were all negative. Abdomen ultrasound scan (US) and abdominal computerized tomography (CT) scan (Fig. 1) showed localized intestinal wall thickening in the right upper abdominal cavity (about 8mm), thickening of the right upper abdominal omentum, extensive lymphadenopathy, multiple abscesses of the liver and spleen, ascites, and right hydrocele. Chest CT showed bilateral upper lobe pneumonia and pleuritis. The patient presented with fever after hospitalization and was administered with intravenous Latamoxef Sodium (70mg/kg/day, every 12h) and subsequently cefoperazone-sulbactam sodium (240mg/kg/day, every 6h) plus linezolid (30mg/kg/day, every 8h) due to suspected bacterial infection. The patient was also treated with IVIG (2g/kg) but the symptoms did not improve. Abdominocentesis was performed which revealed unclear yellowish-green ascetic fluid containing 23453 cells/mm3 with neutrophilic predominance (11913cells/mm3) and protein and glucose concentrations of 46.5 and 58.1 mg/dl respectively. Gram-staining and acid-fast staining tests were all negative. The ascites culture identified B. Contaminans. Therefore, the treatments were switched to meropenem (60mg/kg/day, every 6h) plus linezolid. Following this treatment, he was afebrile and showed improvement in his ascites. Due to concerns of possible underlying primary immunodeficiency, NADPH activity was tested. DHR(dyhydrorhodamine)-1,2,3, can be oxidised to rhodamine-1,2,3, which emits a fluorescent signal detected by the enzyme labelling. The tests showed profound decrease in NADPH activity (139F/ug, reference value 1332-9312F/ug) and relative activity (3%, reference value 31-216%). The patient subsequently developed progressive bilateral cervical lymphadenopathy with low-grade fever. Empirical anti-tuberculosis therapy with rifampicin (10mg/kg/day, in a single dose) was administered which effectively improved the symptoms. Prednisone (1 mg/kg/day, in 2 doses) was administered orally due to persistent intestinal wall thickening, elevated CRP (29-41mg/L), and platelet count (maximum 615×10^9^/L), which decreased the intestinal wall thickening and the platelet count to 368×10^9^/L, while the CRP level to 8mg/L. After 40 days of hospitalization, the patient was discharged on cefdinir (15mg/kg/day, in 3 doses), and was subsequently started on trimethoprim-sulfamethoxazole (sulfamethoxazole 20mg/kg/day in a single dose) and voriconazole (5mg/kg/day in a single dose) prophylaxis along with continuation of linezolid and rifampicin. After discharge, targeted next-generation sequencing analysis identified a nonsense variant in CYBB gene (NM_000397.3; c.603C > A position p. Tyr201*) (Figs. 2, 3, and 4). At a follow-up visit performed after 1 month, prednisone was stopped but the anti-infection therapies were continued. Up to now, his clinical presentation is normal and there is no signs of the disease. Hematopoietic stem cell transplantation (HSCT) was recommended.

## Discussion

Clinically, XLR-CGD is prevalent in men and is carried by women [5]. Recurrent infections are common in patients with CGD. Moreover, CGD may present with granulomas or inflammatory disorders. The infections commonly affect the lungs, skin, lymph nodes, gastrointestinal tract and bone marrow. The common causative pathogens are catalase-positive bacteria and fungi, such as *Staphylococcus aureus, Burkholderia cepacia, Serratia marcescens, Aspergillus fumigatus,* and *Candida albicans* [2].

In the present case, the patient did not show prominent digestive tract symptoms. However, he presented with fever, intestinal wall thickening, and ascites. Culture of the ascetic fluid showed that *B. Contaminans* was positive. The most commonly affected organs in patients with CGD include the gastrointestinal tract (nearly 100%), with inflammatory bowel disease (IBD) accounting for 33% of all cases. Inflammatory and autoimmune complications with the X-linked inheritance pattern are twice as high as those in patients with AR-CGD [6]. In the present patient, invasive enteroscopy was not performed due to the rapid resolution of symptoms. Persistent intestinal wall thickening, elevated levels of CRP, IgG, platelet count, and response to glucocorticoid therapy confirmed the presence of hyperinflammation. In addition, the chest CT showed concealed pneumonia. Hydrocele in the right testes which may have been caused by unclosed sheath process resolved after the initial treatment for about ten days [7].

*Burkholderia *sensu stricto contains the *Burkholderia cepacia complex (Bcc)* and *Burkholderia pseudomallei* group. Both *B. contaminated* and *B. cepacia* belong to the *Bcc.* It is a group of gram-negative bacteria, which are common in the environment, and cause opportunistic infections. The *Bcc* is mostly causing pneumonia in patients with CGD and shows resistance to multiple drugs. *B. contaminated* is sensitive to trimethoprim-sulfamethoxazole, chloramphenicol, minocycline, ceftazidime, piperacillin/tazobactam and carbapenem [8]. It was first reported in patients with cystic fibrosis (CF) and associated with various infections, especially necrotizing pneumonia and the worsening of pulmonary function. Since then, it has been detected in patients undergoing cataract surgery and biliary tract infections, presented as endophthalmitis and sepsis respectively.It is mostly caused by contact with medical devices or contaminated aqueous solutions including nebulized medications, ultrasound gel, nasal spray, lipid emulsion, and hospital water, which may be responsible for hospital outbreaks [9–12]. BCG infections occur frequently in patients with CGD and the patient was given prophylactic anti-tuberculosis treatment (rifampicin).

Due to unusual germs and site of the patient, NADPH activity was tested, and showed a profound decrease in NADPH activity. Further, genome sequencing revealed a de novo nonsense mutation in the coding region of exon 6 of *CYBB*. This mutation shifts tyrosine to a premature termination codon at 201^th^ amino acid (c. 603C > A), resulting in abnormal expression of gp91phox, and its domain is located at the N-terminal. According to recent research, about 61% of mutations in *CYBB* gene occur in the N-terminal domain, mostly fragment deletions and splicing errors [13, 14].

The survival of patients with CGD is closely influenced by production of residual reactive oxygen intermediates (ROIs). Compared with children with AR-CGD, those with XLR-CGD have an earlier disease onset and more severe disease leading to higher mortality. Missense mutations in CYBB can decrease levels of superoxide or gp91phox expression. In contrast, nonsense mutations inhibit the production of superoxide and protein expression, thereby decreasing the survival rate [15, 16].

Anti-infective therapy can significantly improve the quality of life and survival rate of CGD patients. Subcutaneous injection of interferon-γ decreases the risk of infection, especially in patients who acquire infections while on prophylactic antibiotics and those infected with tuberculosis [17]. Patients with CGD have excessively high levels of cytokines, such as TNF-α, IL-1, IL-8, which predispose them to hemophagocytic lymphohistiocytosis (HLH) when infected. The incidence of inflammatory complications in patients with XLR-CGD is twice as high as that in patients with AR-CGD [6, 18]. Appropriate anti-inflammatory agents, mainly corticosteroids, can significantly improve the prognosis of patients with CGD, and do not appear to increase the bacterial infection risk [3]. The recommended initial dose of prednisone is 1 mg/kg daily and to be given for an average of 2-3 weeks before being tapered over several months [19]. Biological disease modifying antirheumatic drug (bDMARDs), such as anti-TNF-a monoclonal antibodies, recombinant IL-1receptor-targeted antagonist and IL-23 antagonist have been used, but there are increased risks of infection. HSCT remains the best curative option for CGD. The major problem is the risk of infection and graft-versus-host disease (GVHD). Lower risk patients may be best treated by HSCT. For high-risk patients and patients who have no well-matched donor, gene therapy may be considered. Although preliminary results using lentiviral vectors are fairly encouraging, gene therapy still faces many challenges [3, 20].

## Conclusion

We suggest that the presence of *B. Contaminans* in patients with CGD should be explored. Early diagnosis is crucial to facilitate effective treatment.

## Data Availability

Not applicable.

## Abbreviations

CGD: Chronic granulomatous disease
*B.cepacia*: *Burkholderia cepacia*
*B.cepacia*: *Burkholderia cepacia*
*B. Contaminans*: *Burkholderia contaminans*
*Bcc*: *Burkholderia cepacia complex*
NADPH: Nicotinamide adenine dinucleotide phosphate
BCG: Bacillus Calmette-Guerin
CRP: Reactive protein
ESR: Erythrocyte rate sedimentation
CT: Computerized tomography
US: Ultrasound scan
IGRA: Interferon-gamma release assay
IBD: Inflammatory bowel disease
WBC: White blood cell count
MCV: Mean Corpuscular Volume
MCH: Mean Corpuscular Hemoglobin
MCHC: Mean corpuscular hemoglobin concentration
HSCT: Hematopoietic stem cell transplantation
GVHD: Graft-versus host disease;CF:cystic fibrosis
ROIs: Reactive oxygen intermediates
HLH: Hemophagocytic lymphohistiocytosis
DHR-1,2,3: Dyhydrorhodamine-1,2,3
